# Wearable Triboelectric Nanogenerator with Ground-Coupled Electrode for Biomechanical Energy Harvesting and Sensing

**DOI:** 10.3390/bios13050548

**Published:** 2023-05-15

**Authors:** Kangyu Su, Xiaobo Lin, Zhangwei Liu, Yun Tian, Zhengchun Peng, Bo Meng

**Affiliations:** Key Laboratory of Optoelectronic Devices and Systems of Ministry of Education and Guangdong Province, College of Physics and Optoelectronic Engineering, Shenzhen University, Shenzhen 518060, Chinazcpeng@szu.edu.cn (Z.P.)

**Keywords:** triboelectric nanogenerator, wearable sensor, biomechanical energy harvesting, human motion monitoring

## Abstract

Harvesting biomechanical energy for electricity as well as physiological monitoring is a major development trend for wearable devices. In this article, we report a wearable triboelectric nanogenerator (TENG) with a ground-coupled electrode. It has a considerable output performance for harvesting human biomechanical energy and can also be used as a human motion sensor. The reference electrode of this device achieves a lower potential by coupling with the ground to form a coupling capacitor. Such a design can significantly improve the TENG’s outputs. A maximum output voltage up to 946 V and a short-circuit current of 36.3 μA are achieved. The quantity of the charge that transfers during one step of an adult walking reaches 419.6 nC, while it is only 100.8 nC for the separate single-electrode-structured device. In addition, using the human body as a natural conductor to connect the reference electrode allows the device to drive the shoelaces with integrated LEDs. Finally, the wearable TENG is able to perform motion monitoring and sensing, such as human gait recognition, step count and movement speed calculation. These show great application prospects of the presented TENG device in wearable electronics.

## 1. Introduction

With the rapid development of wearable devices, finding constant power for wearable electronics is a matter of urgency. Conventional power sources suffer disadvantages in limited lifespan and environmental pollution. Harvesting energy from the environment to power these devices is a viable strategy [1,2,3,4]. The human body can generate a lot of biomechanical energy during movement [5,6,7,8], which can be harvested by micro-energy harvesting technology [9,10]. The triboelectric nanogenerator (TENG) was first reported in 2012 by Wang’s group [11,12,13,14]. Based on the coupling of contact electrification and electrostatic induction, it can convert mechanical energy into electrical energy, thus realizing energy harvesting and active sensing [15,16,17,18]. With the advantages of high energy conversion efficiency, rich material selection and a simple preparation process, it is an excellent choice for wearable electronics [19,20,21,22].

The movements of human feet generally have high intensity, which is an ideal choice for energy harvesting, as proposed in previous studies [23,24,25,26,27]. A wearable TENG used on the feet should meet some requirements: flexible, durable and comfortable. For the electrode layer of a TENG, it is necessary to select materials with better fatigue characteristics. The microstructure of the friction layer surface has been proven to influence the performance of a TENG significantly [28,29,30]. The use of a single-electrode-structured TENG in wearable devices has been reported [31,32,33]. This can effectively avoid the limitations of requiring a spacer. However, how to effectively improve the output performance of single-electrode-mode devices is a great challenge. Furthermore, the monitoring of human motion through self-powered wearable devices is also an important trend of research [34,35,36,37,38,39,40,41].

Herein, we report a wearable TENG with a ground-coupled electrode. The wearable TENG does not require a spacer to provide an initial vertical gap. It consists of two single-electrode devices attached to each sole. Each step generated by the foot’s movement is able to drive the wearable TENG for biomechanical energy harvesting. During the motions, one of the two devices always serves as a ground-coupled electrode. Such a design can significantly improve the output performance compared to the device without grounded coupling. By using the human body as a natural conductor instead of external wires to connect the separate devices, the practicality of the wearable TENG is improved. The application of self-powered luminous shoelaces with integrated LEDs is also demonstrated. Furthermore, gait recognition to judge the movements of the human body is realized by monitoring the TENG’s output voltage generated by human motion. 

## 2. Materials and Methods

### 2.1. Design and Fabrication of the Wearable TENG

**Figure 1:**
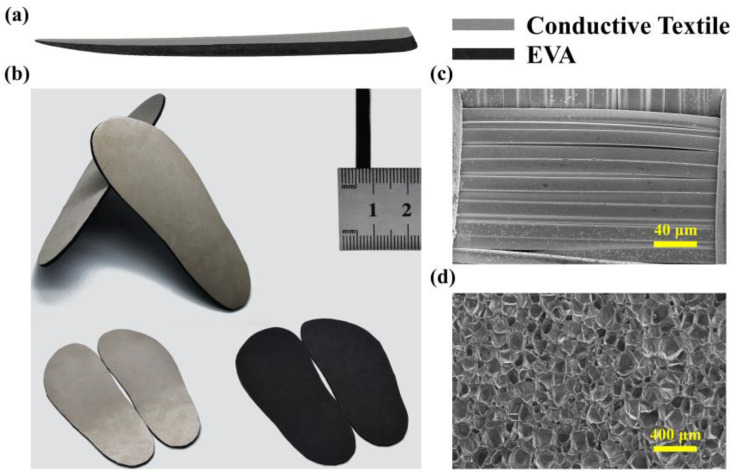
Schematic and photographs of the wearable TENG. (**a**) Schematic diagram of the wearable TENG. (**b**) Photographs of the wearable TENG. (**c**) SEM image of the conductive textile. (**d**) SEM image of the EVA foam.

Figure 1a shows a schematic of the proposed wearable TENG. Due to the simple structure of the device, it is easy to fabricate. The conductive textile tape used here mainly consists of polyester fiber with acrylic conductive adhesive. Moreover, the fiber is coated with metal, copper and nickel, meaning it has good conductivity. Since we use EVA foam with adhesive on one side, the whole fabrication of the wearable TENG is simple and rapid. The following steps can be followed to fabricate the device: Cut the EVA foam and conductive textile tape into the needed size. Then, attach the tape to the surface of the EVA foam. For the integrated TENG device, the exposed side of the EVA is used as the friction layer and the conductive textile tape is used as the electrode. Benefitting from excellent flexibility, high conductivity and wear resistance, conductive textile tape is an excellent choice for wearable electrode material. Photographs of the sole-sized device are shown in Figure 1b. The area of sole can be regarded as the effective contact area of the TENG. In this work, we use a pair of common-size 42 (CN) shoes, and the effective area of the TENG is about 208 cm^2^. The thickness of the EVA foam is approximately 4 mm, and in comparison, the conductive textile tape is very thin. Therefore, almost all of the thickness of the device concentrates on the EVA foam layer. The wearable TENG integrated with 4 mm thick EVA foam has the most excellent output performance, as shown in Figure S1 (Supplementary Materials). Both its output voltage and short-circuit current are the highest compared to the devices with 3 mm and 5 mm thick foam. It should be noted that devices worn on the left and right foot have the same data characteristics. Due to the difference in the strength of human feet movement, it will lead to a slight difference in the test data for the two feet. Figure 1c,d show the SEM images of the conductive textile and EVA foam. We found that the EVA foam has porous microstructure, which can increase the contact area between the friction layer and floor. More scale SEM images are shown in Figure S2.

### 2.2. Working Mechanism of the Wearable TENG

**Figure 2:**
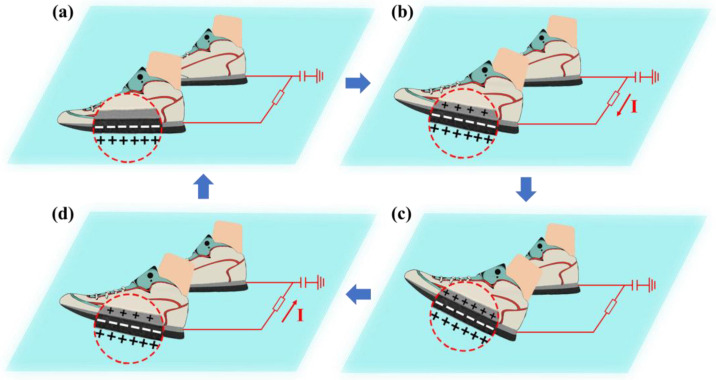
Working mechanisms of the wearable TENG. (**a**) Both feet on the floor. (**b**) One foot is lifting while the other foot is on the floor. (**c**) One foot lifted to the highest with the other foot on the floor. (**d**) One foot is falling while the other foot is on the floor.

Working mechanisms of the wearable TENG are illustrated in Figure 2. The wearable TENG system contains two separate single-electrode devices. The two devices are attached separately to the sole and connected by a wire. Such a combination of devices ensures that there is always one foot entirely in contact with the floor, providing a good grounding during human walking. This is the essential configuration of the wearable TENG system. The combination of dual devices constitutes the wearable TENG system with ground-coupled electrode to achieve structural optimization and performance enhancement. The wearable TENG works based on triboelectric effect and electrostatic induction. At the initial stage shown in Figure 2a, when both feet stand on the floor with no movement, the devices attached to the sole are in full contact with the floor. Compared with the floor, EVA foam tends to attract electrons. Therefore, the electrons are injected into the EVA foam from the floor, and, meanwhile, the floor gains an equal amount of positive charge. When a single foot lifts up (Figure 2b), the surface of the EVA foam separates from the floor. At the same time, the other foot still maintains contact with the floor, so this foot is grounded during the process. Furthermore, it provides a higher electric potential to form a potential difference in the entire circuit. For returning to electrostatic equilibrium state, the potential difference drives the electrons to flow from the lifted foot to the grounded foot, which generates a current. The current can power on external electronics. Once the system reaches the electrostatic equilibrium state, no current flows through the external circuit (Figure 2c). Similarly, as the lifted foot gradually falls, the EVA foam and floor become closer. As a result, a reversed current is generated from the falling foot to the other grounded foot (Figure 2d). Generally, the wearable TENG system with ground-coupled electrode alternately generates current in the external circuit during human walking. The equivalent circuit of this wearable TENG is shown in Figure S3a.

## 3. Results and Discussions

### 3.1. Characteristics of the Wearable TENG

**Figure 3:**
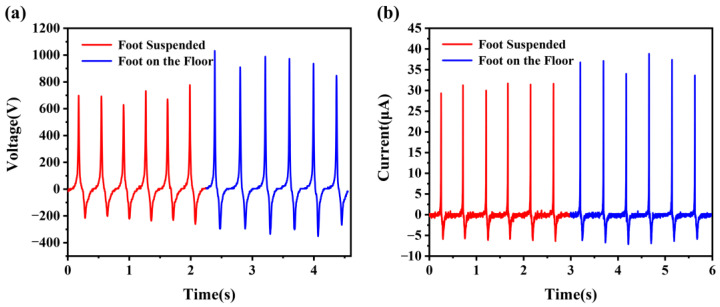
An output performance comparison of the wearable TENG with foot suspended or on the floor. (**a**) Output voltage and (**b**) short-circuit current of the TENG in a sitting position with a single foot suspended or on the floor when the other foot is repeatedly stepping on the floor.

We set up an experiment to demonstrate that the wearable TENG system with a ground-coupled electrode has significantly improved output performance, as shown in Figure 3. In this experiment, we take a sitting position to test the output of two situations. One foot is always suspended in the air, and the other foot is repeatedly stepping on the floor (About 2.5 Hz). The average values of output voltage and short-circuit current of the wearable TENG are 698 V and 30.8 μA (Figure 3a,b), respectively. Then, when one foot is always in contact with the floor, and the other foot is repeatedly stepping on the floor (About 2.5 Hz), the average values of output voltage and short-circuit current of the wearable TENG are 946 V and 36.3 μA (Figure 3a,b), respectively, which are 35.5% and 17.9% higher than the first situation. It can be further indicated that the output performance of the wearable TENG with one of the sole devices contacting the floor as the reference electrode for coupling to the ground is significantly improved. It proves that the proposed structural optimization is effective. The energy harvesting system with a ground-coupled electrode improves the overall output performance of the TENG, and it compensates for the low output of the common single-electrode mode TENG.

**Figure 4:**
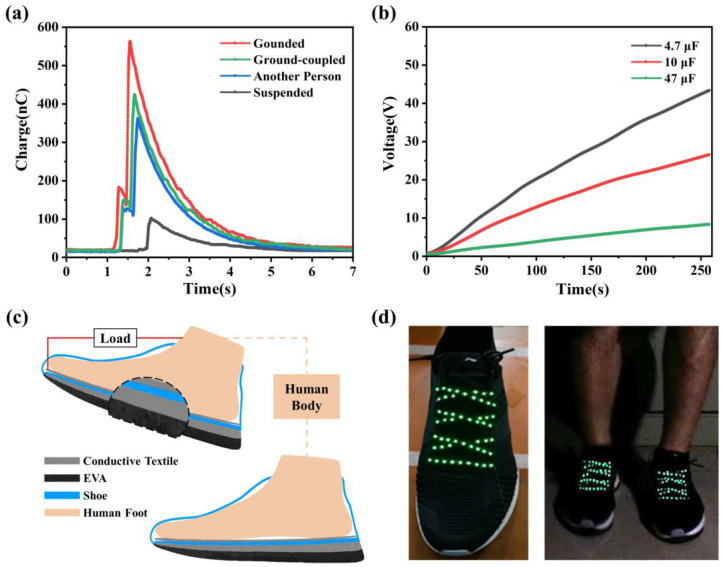
Performance of the capacitor charging and application of the wearable TENG. (**a**) Comparison of the quantity of transferred charge in one step (capacitance is 0.1 μF). (**b**) Voltage curves of charging capacitors using the wearable TENG (4.7 μF, 10 μF, 47 μF). (**c**) Structure diagram of the optimized device. (**d**) The output power obtained from the wearable TENG is used for lighting up the LEDs integrated on shoelaces.

We characterized the output performance of the wearable TENG with different reference electrodes [32], as shown in Figure 4a. The quantity of transferred charge is measured by charging a 0.1 μF capacitor via a full-wave rectifier bridge. Firstly, we utilize a single device (reference electrode suspended) to perform the test and find that the quantity of transferred charge in one footstep is only 100.8 nC. However, when we test the wearable TENG system with a ground-coupled electrode, the quantity of transferred charge can easily reach up to 419.6 nC. The output performance also increases over 316.3% compared to the single device with a suspended reference electrode. For the other two experiments, we chose another person and the real ground as the reference electrode. The quantities of transferred charge were 361.5 nC and 561.0 nC, respectively. Although the quantity of transferred charge of the presented wearable TENG was 25.2% lower than that of the device with the real ground as a reference electrode, it was still a considerable output, especially compared with other reference electrode experiments. The wearable TENG cannot connect to the real ground as a reference electrode in real time, so the ground-coupled electrode is a better solution. Then, we tested the performance of capacitor charging for the wearable TENG. We used the wearable TENG to charge different capacitors via a full-wave rectifier bridge, as shown in Figure 4b. Thus, 4.7 μF, 10 μF and 47 μF capacitors were charged to 43 V, 27 V and 9 V, respectively. These data show that the wearable TENG can meet the power consumption of common electronic components in wearable electronics. In addition, the power density curve under different external loading resistance is shown in Figure S4. The maximum power output is 19.68 mW at the optimum external load resistance of about 33 MΩ. Therefore, the power density can be calculated as 0.94 W/m^2^.

The wearable TENG system may have some limitations in practical application. When the separate sole devices are connected by external wire to form a ground-coupled electrode, it will reduce the integration of the whole system. The wearable TENG can be suitably optimized to meet the requirements in real applications. First, an additional layer of conductive textile tape is attached to the bottom of the shoe as an electrode, as shown in Figure 4c. When charge is generated by the contact–separation motion between the EVA foam and the floor, an electric field is formed on the electrode surface at the back of the device accordingly. At the same time, based on the electrostatic induction effect, the electrode inside the shoe will be affected by the outside of the device electrode to generate induced charge. To avoid the introduction of external wires, the human body is used as a natural conductor to connect the two separate sole devices. We connect the electrode inside the shoe to the load and connect the other end of the load through the human body to another device as a reference electrode, forming a complete circuit. When the human walks, there is a significant potential difference between the loads, satisfying the conditions for driving the load to work. The equivalent circuit of the wearable TENG with the human body used as the conductor is shown in Figure S3b. As shown in Figure 4d, the LED strip integrated on the shoelaces was lit by the above system. A detailed demonstration of the process can be seen in the Supporting Video S1.

### 3.2. Application on Human Motion Monitoring

**Figure 5:**
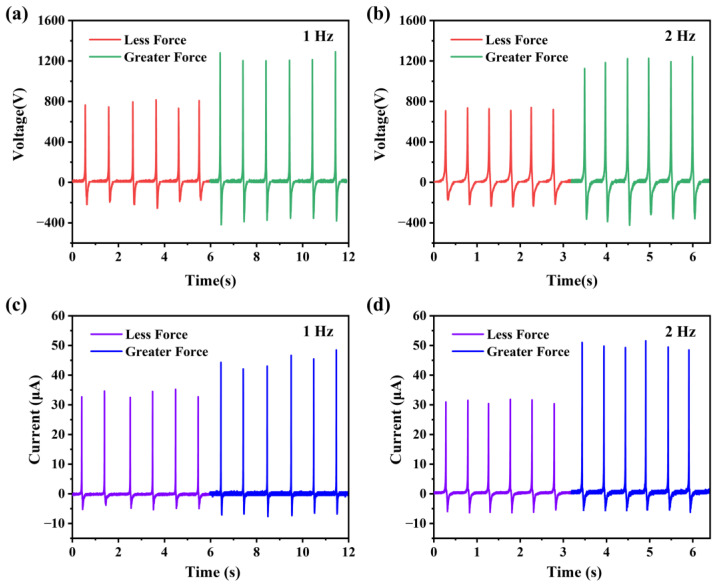
The output voltage and short-circuit current comparison of the wearable TENG. Output voltage at (**a**) 1 Hz and (**b**) 2 Hz when stepping with less or greater force. Short-circuit current at (**c**) 1 Hz and (**d**) 2 Hz when stepping with less or greater force.

The output performance of the wearable TENG is mainly characterized under different frequencies and different forces when a single foot is repeatedly stepping on the floor, and the other foot is keeping in contact with the floor. Figure 5 shows the output voltage and short-circuit current comparison of the wearable TENG. Here, we are stepping on the floor with two force levels at 1 Hz and 2 Hz, respectively. The average force applied to the TENG during contact and separation is about 390 N for “less force” and 695 N for “greater force”. Since the force is related to the weight (in this work, the wearer’s weight is 71 kg) and motion of the wearer, we also measured the acceleration curve of the wearer’s foot during the motions (Figure S5). The maximum acceleration measured under less force is about 2 g to 2.5 g, and it is 4 g to 4.5 g under greater force. Figure 5a indicates that when stepping on the floor lightly at a frequency of 1 Hz, the average output voltage is 774 V. When testing the system with greater force at the same frequency, the average output voltage is 1231 V. After that, we try to step on the floor at two force levels and measure voltage at a frequency of 2 Hz. As shown in Figure 5b, under less force, the average output voltage is 720 V. When stepping on the floor heavily, the average output voltage is 1197 V. 

Moreover, an output short-circuit current comparison of the wearable TENG is shown in Figure 5c,d, which exhibits the same data characteristics. Figure 5c indicates that, when stepping on the floor lightly at a frequency of 1 Hz, the average short-circuit current is 33.4 μA. When testing the system with greater force at the same frequency, the average short-circuit current is 45.4 μA. After that, we try to step on the floor at two force levels and measured the current at a frequency of 2 Hz. As shown in Figure 5d, under less force, the average short-circuit current is 31.0 μA. When stepping on the floor heavily, the average short-circuit current is 49.9 μA. These data show that the amplitude of the output is not sensitive to the frequency of motion when the TENG is applied with the same force. That is because the velocity and force applied to the TENG during contact and separation changed only a little with the frequency. This is very different from the simple harmonic stimuli applied by a linear motor. On the other hand, the frequency and strength of human walking can be judged by the change in frequency and amplitude of the output waveform. Thus, it is able to achieve a preliminary analysis of gait. We can then calculate the wearer’s speed or distance. In addition, the durability test of the TENG device was conducted. The TENG was stimulated by a linear motor at a frequency of 1 Hz for 7000 repeated cycles and showed a stable output under long-term operation (Figure S6). 

**Figure 6:**
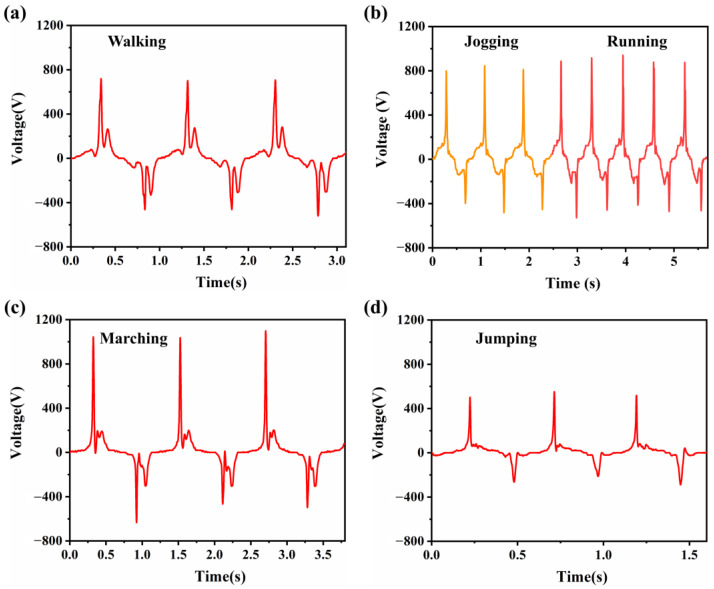
Waveforms comparison under different motions. (**a**) The voltage waveform of walking. (**b**) The voltage waveform of jogging and running. (**c**) The voltage waveform of marching. (**d**) The voltage waveform of jumping.

To further investigate the recognition of human motion via the wearable TENG, we measured the waveforms of five different actions: walking, jogging, running, marching and jumping, as shown in Figure 6. In the process of walking, while the raised foot is about to land on the floor, the heel usually touches the floor first (greater force), and then the front part of the foot contacts the floor (less force). Hence, these movements result in two positive peaks in the output waveform, one large (709 V) and one small (274 V) (Figure 6a). Jogging and running are relatively similar to walking, except that the movement is much faster, and the contact force is greater. Therefore, only one positive peak and one negative peak appear in the waveform (816 V and −450 V for jogging, 897 V and −473 V for running) (Figure 6b). It is worth noting that jogging shows differences in frequency and intensity compared to normal running. It shows up in the output waveform with significant differences in frequency and voltage peaks. Thus, the wearable TENG can capture the differences between these two different gait patterns as well. Marching is a special movement. The lifted foot should land heavily parallel to the floor. Because the sole is not exactly flat, it may cause a much larger positive peak (1060 V) and some smaller peaks (199 V) (Figure 6c). As for jumping, there is an obvious drop in peak output (523 V) compared to other waveforms of human motion (Figure 6d). The reason is that the working mode of the wearable TENG is changed in the motions of jumping. Both feet lift off the ground at the same time during the jump. In such a case, there is no electrode being ground coupled. Thus, the TENG changes into a separate single-electrode mode with a suspended reference electrode. As discussed above, the outputs of the wearable TENG would be reduced when the reference electrode changes from ground-coupled state to suspended state. The output waveforms corresponding to the gait states are distinctly different. We can achieve gait recognition according to the output signal of the wearable TENG. Based on the experiments mentioned above, we prove that the wearable TENG system also has the potential to recognize human motion, especially movement speed, step count, step frequency and gait.

## 4. Conclusions

In summary, through structural optimization, a wearable TENG with a ground-coupled electrode was designed to harvest biomechanical energy and monitor human motion. Compared with a simple single-electrode-structured device, we prove that the wearable TENG system with a ground-coupled electrode is efficient for improving the output performance. Notably, its maximum output voltage and current can reach up to 946 V and 36.3 μA, which are increased by 35.5% and 17.9%, respectively. Thus, 419.6 nC charge can be transferred in one step, which is over four-times larger than the device without grounding. In addition, using the human body as a natural conductor makes the entire system highly integrated and allows for fully wearable applications. The wearable TENG can not only provide a considerable power generation but also serve as a self-powered sensor for human motion monitoring. Through analyzing the output waveforms, the wearable TENG can achieve gait recognition and detect the contact strength between feet and the floor. Hence, we believe the wearable TENG has great potential in monitoring human motion and other wearable applications.

## Experimental Methods

### Characterization of the Device 

The voltage of the TENG was measured using a handheld oscilloscope (SIGLENT, Shenzhen, China, SHS806) with a probe impedance of 100 MΩ. The short-circuit current was measured via a low-noise current preamplifier (Stanford, Sunnyvale, CA, USA, SR570). The morphology of the conductive textile and EVA foam was observed using an SEM (Hitachi, Tokyo, Japan, SU8010).

## Data Availability

The experimental data are contained within the article and the Supplementary Materials.

## Supplementary Materials

The references in Supplementary Materials were cited in Refs. [34,38,41,42,43,44,45,46,47,48,49]. The following supporting information can be downloaded at: https://www.mdpi.com/article/10.3390/bios13050548/s1, Figure S1: Output performance comparison of the wearable TENG using EVA triboelectric layers with different thicknesses; Figure S2: SEM images of the conductive textile and the EVA foam; Figure S3: Schematic of the equivalent circuits of the wearable TENG; Figure S4: Power density curve under different external loading resistance; Figure S5: Acceleration curve of the tester’s foot during the motions; Figure S6: Durability test of the TENG; Table S1: Comparison of different TENG devices for biomechanical energy harvesting and human motion monitoring; Supporting Video S1: The wearable TENG lighting up the LED strip integrated in the shoelace during normal walking.
