# Supplementary material for: Wearable Triboelectric Nanogenerator with Ground-Coupled Electrode for Biomechanical Energy Harvesting and Sensing
Source: Biosensors (Basel). 2023 May 15;13(5):548. doi: 10.3390/bios13050548 (PMC10216270; doi:10.3390/bios13050548)
Supplement: Supplementary file 1 [file biosensors-13-00548-s001.zip › Supplementary Materials.pdf]

biosensors

Supplementary Materials

Wearable Triboelectric Nanogenerator with Ground-Coupled Electrode for Biomechanical Energy Harvesting and Sensing

Kangyu Su †, Xiaobo Lin †, Zhangwei Liu, Yun Tian, Zhengchun Peng and Bo Meng *

Key Laboratory of Optoelectronic Devices and Systems of Ministry of Education and Guangdong Province, College of Physics and Optoelectronic Engineering, Shenzhen University, Shenzhen 518060, China; zcpeng@szu.edu.cn (Z.P.)

* Correspondence: bomeng@szu.edu.cn

† These authors contributed equally to this work.

Citation: Su, K.; Lin, X.; Liu, Z.; Tian, Y.; Peng, Z.; Meng, B. Wearable Triboelectric Nanogenerator with Ground-Coupled Electrode for Biomechanical Energy Harvesting and Sensing. *Biosensors* **2023**, *13*, 548. https://doi.org/10.3390/bios13050548

Received: 30 March 2023
Revised: 10 May 2023
Accepted: 11 May 2023
Published: 15 May 2023

Copyright: © 2023 by the authors. Licensee MDPI, Basel, Switzerland. This article is an open access article distributed under the terms and conditions of the Creative Commons Attribution (CC BY) license (https://creativecommons.org/licenses/by/4.0/).

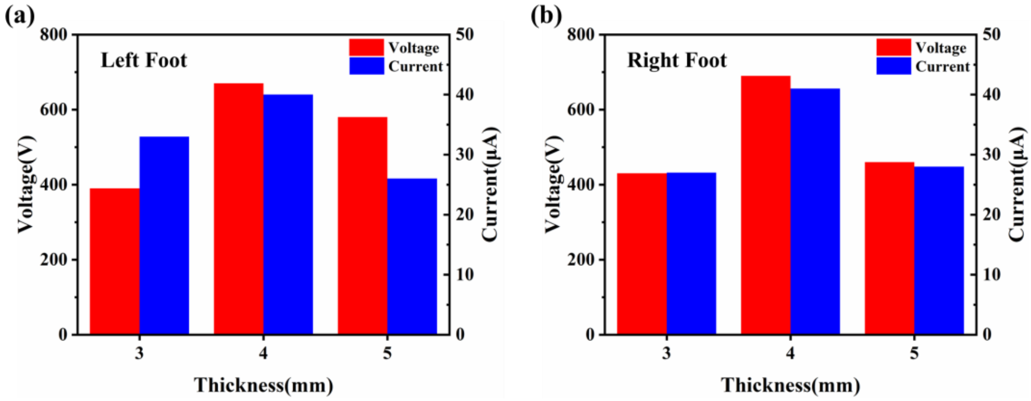

Figure S1. Output performance comparison of the wearable TENG using EVA triboelectric layers with different thicknesses. (**a**) Output comparison of the wearable TENG worn on the left foot. (**b**) Output comparison of the wearable TENG worn on the right foot.

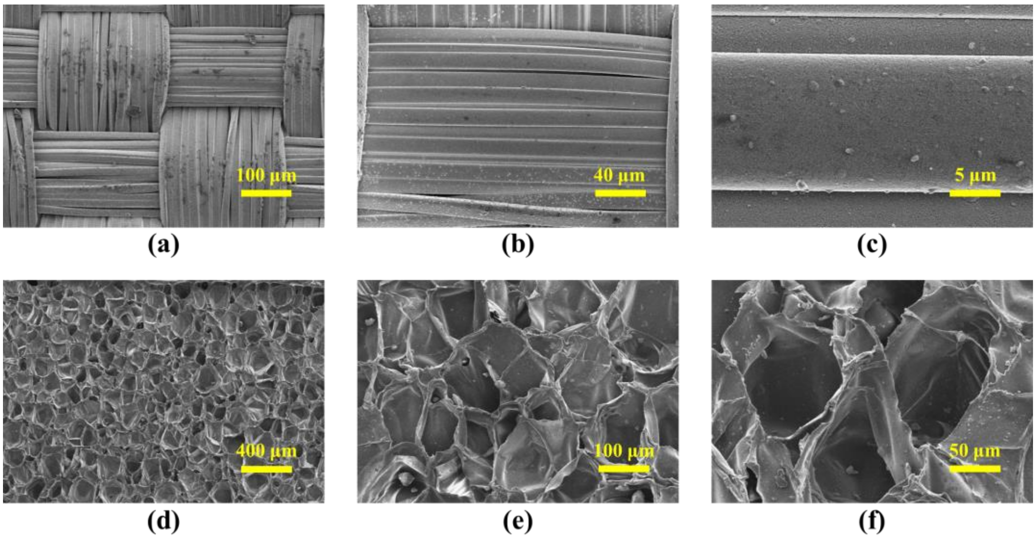

Figure S2. SEM images of the conductive textile and the EVA foam. (**a-c**) SEM images in different scales of the conductive textile. (**d-f**) SEM images in different scales of the EVA foam.

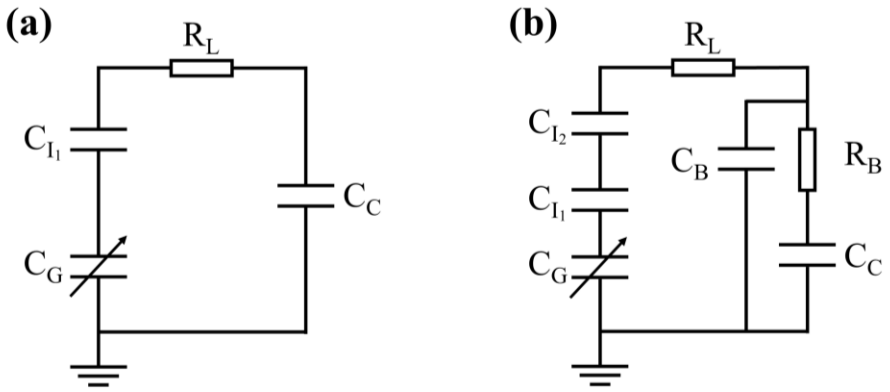

Figure S3. Schematic of the equivalent circuits of the wearable TENG. (**a**) Equivalent circuit diagram of the TENG with an external wire. (**b**) Equivalent circuit diagram of the TENG with human body as a natural conductor.

The equivalent circuit of the device can be simplified to **Figure S3**. In which R_L corresponds to the load resistor, C_G represents the capacitance between the friction surface of sole and the ground, C_C represents the capacitance between ground-coupled electrode and ground, C_{I1} represents the capacitance between the TENG's electrode and the friction surface, C_{I2} is the capacitance between the electrodes inside and outside the shoe (when human body is used as a natural conductor instead of an external wire), C_B is the capacitance between the ground and human body, and R_B is the resistor of human body. When one foot keeps contact with the ground, the device on this foot forms a ground-coupled electrode. And the capacitance between the electrode and ground (C_C) is much larger than the capacitance in the state where the foot is suspended. Therefore, it provides a greater potential difference for charge transfer.

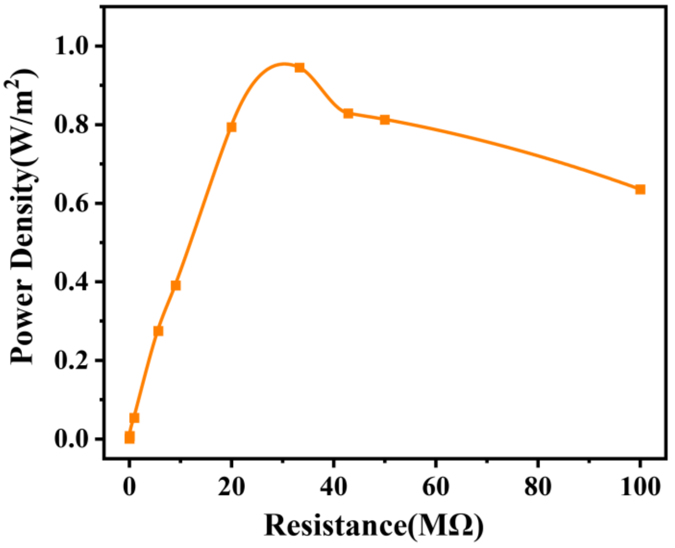

Figure S4. Power density curve under different external load resistance.

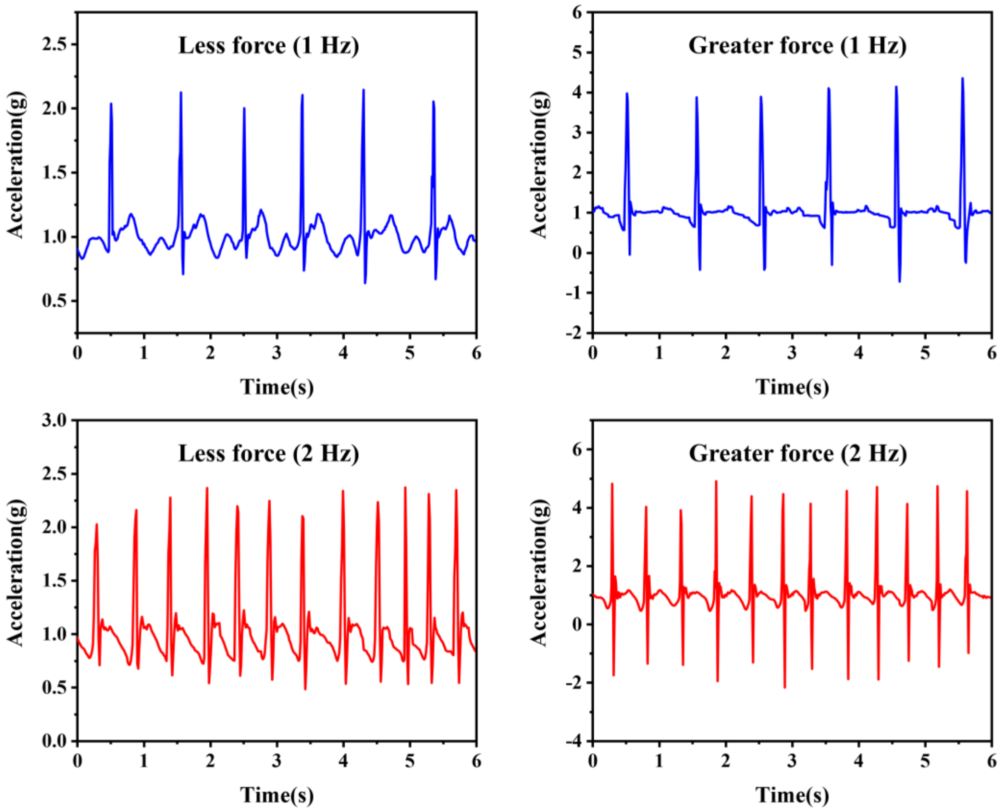

Figure S5. Acceleration curve of the tester's foot during different motions.

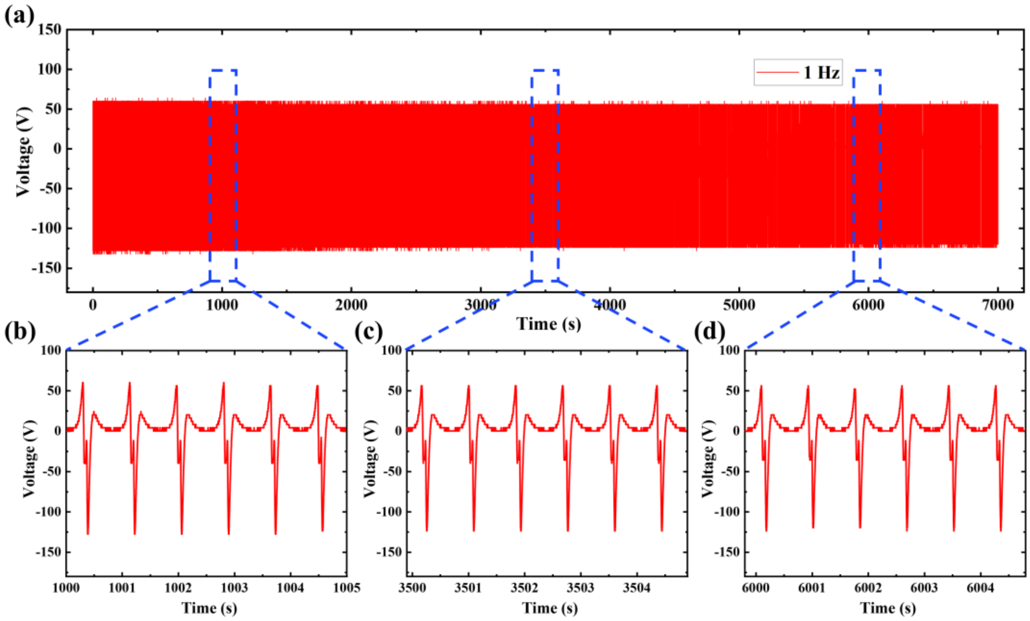

Figure S6. Durability test of the TENG.

Table S1. Comparison of different TENG devices for biomechanical energy harvesting and human motion monitoring.

Materials	Structural Features	Output Performance	Location of Installation	Applications	
rubber and physiological saline	rubber surface nanostructures	0.33 mW/m^2	calf and other human body parts	gait pattern detection and identity recognition	[38]
laser-induced graphene foam	3D porous network structure	92.1 mW/m^2	chest, finger, wrist, shoe soles	motion monitoring and health monitoring	[42]
PTFE and PET	modified ratchet–pawl structure	22.004 W/m^3	lower-limb	gait abnormity detection and lower-limb motion monitoring	[43]
copper-nickel fabric and $BaTiO_3$ doped PDMS	multi-layer corrugated structure	4860 mW/m^2	Insoles and carpets	people stream monitoring and falls detection	[41]
PAN@ZIF-8 nanofibers and PTFE	rough surface supporting structure	1910 mW/m^2	home appliances	monitor and control home appliances	[44]
chitosan and silk fibroin film，PTFE	sandwich structure	268.8 mW/m^2	shoe soles and human body parts	human motion monitoring	[34]
PDMS and PEDOT:PSS	electrode with wrinkled surface structure	4.06 mW/m^2	elbow and joint	bending angle of elbow and joint monitoring	[45]
P(VDF-TrFE) and PA66	woven fabric structure	93 mW/m^2	gloves	finger movements detection	[46]
PTFE and aluminum foil	concave-convex supporting structure	0.686 mW/m^2	shoe heels	gait and stability of multiple human motions monitoring	[47]
PEDOT:PSS and PZT	piezoelectric and triboelectric hybrid structure	110 mW/m^2	socks	walking pattern recognition and motion tracking	[48]
Kevlar fiber and conductive shear-stiffening gel	protective and impact kinetic energy-harvesting properties	5.3 mW/m^2	Elbow, knee,	sports protection and foot motion recognition	[49]
EVA and conductive textile	ground-coupled electrode	940 mW/m^2	shoe soles	human gait recognition, motion monitoring	**This Work**

Supporting Video S1. The wearable TENG lighting up the LED strip integrated in the shoelace during normal walking.
